# Site-selective spectroscopy with depth resolution using resonant x-ray reflectometry

**DOI:** 10.1038/s41598-017-12642-7

**Published:** 2017-10-23

**Authors:** J. E. Hamann-Borrero, S. Macke, B. Gray, M. Kareev, E. Schierle, S. Partzsch, M. Zwiebler, U. Treske, A. Koitzsch, B. Büchner, J. W. Freeland, J. Chakhalian, J. Geck

**Affiliations:** 1 Leibniz Institute for Solid State and Materials Research, IFW Dresden, 01171 Dresden, Germany; 2 Quantum Matter Institute, University of British Columbia, 2355 East Mall, Vancouver, V6T 1Z4 Canada; 3 Max Planck Institute for Solid State Research, Heisenbergstraße 1, 70569 Stuttgart, Germany; 4 Department of Physics, University of Arkansas, Fayetteville, Arkansas 70701 USA; 5 Department of Physics and Astronomy, Rutgers University, Piscataway, New Jersey 08854 USA; 6 Helmholtz-Zentrum Berlin für Materialien und Energie, Albert-Einstein-Str. 15, D-12489 Berlin, Germany; 7 Institut für Festkörper- und Materialphysik, TU Dresden, D-01062 Dresden, Germany; 8 Advanced Photon Source, Argonne National Laboratory, Argonne, Illinois 60439 USA

## Abstract

Combining dissimilar transition metal oxides (TMOs) into artificial heterostructures enables to create electronic interface systems with new electronic properties that do not exist in bulk. A detailed understanding of how such interfaces can be used to tailor physical properties requires characterization techniques capable to yield interface sensitive spectroscopic information with monolayer resolution. In this regard resonant x-ray reflectivity (RXR) provides a unique experimental tool to achieve exactly this. It yields the element specific electronic depth profiles in a non-destructive manner. Here, using a YBa_2_Cu_3_O_7−*δ*_ (YBCO) thin film, we demonstrate that RXR is further capable to deliver site selectivity. By applying a new analysis scheme to RXR, which takes the atomic structure of the material into account, together with information of the local charge anisotropy of the resonant ions, we obtained spectroscopic information from the different Cu sites (e.g., chain and plane) throughout the film profile. While most of the film behaves bulk-like, we observe that the Cu-chains at the surface show characteristics of electron doping, whereas the Cu-planes closest to the surface exhibit an orbital reconstruction similar to that observed at La_1−*x*_Ca_*x*_MnO_3_/YBCO interfaces.

## Introduction

Recent developments in the atomic layer by layer synthesis of transition metal oxide materials and the possibility to put dissimilar materials face-to-face at an interface has provided a vast playground for exciting physics^1–3^. Prominent examples are the formation of a 2D electron gas at the LaAlO_3_/SrTiO_3_ interface^4^ and the observation of superconductivity at interfaces of non-superconducting copper oxides^1,3,5,6^. The nature of these new phenomena has been addressed to be closely related to various reconstruction mechanisms, affecting the charge, spin and orbital states of the TM site at the interface.

For example, the distribution of electrons among local orbitals of the TM site (orbital occupation) has been reported to be strongly modulated at hetero-interfaces. In bulk YBCO the holes in the superconducting CuO_2_ planes are known to be confined to the *xy*-plane occupying the Cu $3{d}_{{x}^{2}-{y}^{2}}$ orbitals^7^. However, at an interface with ferromagnetic La_2/3_Ca_1/3_MnO_3_(LCMO), holes have been found to partially occupy the $3{d}_{3{z}^{2}-{r}^{2}}$ orbital, which is oriented perpendicular to the interface^8–10^. This orbital reconstruction appears to drastically change the electronic properties of the interface with respect to the bulk. Charge from the manganite side is transferred into the cuprate through the Cu-O-Mn bond and induces a magnetic moment on the Cu site at the interface. This moment couples antiferromagnetically with Mn and locally destroys the superconducting state^8,9^. Similar situations have been observed at other cuprate/perovskite interfaces such as (CaCuO_2_(CCO)/STO^11^ and La_1−*x*_Sr_*x*_MnO_3_(LSMO)/CCO^12^) and has also been ascribed to the hybridization of $3{d}_{3{z}^{2}-{r}^{2}}$ orbitals of Cu across the interface via the apical oxygen.

In this situation examining spatial modulations in the electronic system with atomic depth resolution is of paramount importance. Not only for understanding the physics behind these novel phenomena, but also for the design and manipulation of material properties towards functional devices and applications.

Notwithstanding, while various experiments can provide structural profiles of films and heterostructures^13–23^, real depth-resolved spectroscopy remains to be a major experimental challenge, especially since these interfaces are normally buried deep below the sample surface. In this regard, RXR provides a unique experimental tool to study such effects. It is a state of the art technique that combines elastic scattering (reflectometry) with spectroscopy (X-ray absorption), hence allowing to acquire element specific information on electronic properties with monolayer depth resolution^24,25^. In the case of 3*d*-TMOs, information on the relevant 3*d* states is obtained by performing RXR measurements at the *L*_2,3_ edges (i.e., 2*p* → 3*d*) of the TM ion. By judiciously selecting the photon polarization and energy (*hν*) one gets access to information about the different electronic degrees of freedom of the resonant site. Regarding the magnetic degrees of freedom, for instance, sensitivity to magnetism and magnetic depth profiles is provided by the strong x-ray magnetic circular dichroism at the transition metal *L*-edges. (see ref.^26^ and references therein for examples of magnetic RXR). In contrast, information about the spatial charge distribution (orbitals) is obtained from dichroic effects observed using linearly polarized light. In this regard only few works have succeeded to extract information about orbital occupancies and reconstruction profiles from RXR^27–29^, showing RXR to be sensitive enough to resolve fine orbital changes of the order of ~3–5%. Recently, valence state profiles have also been determined^30^.

**Figure 1:**
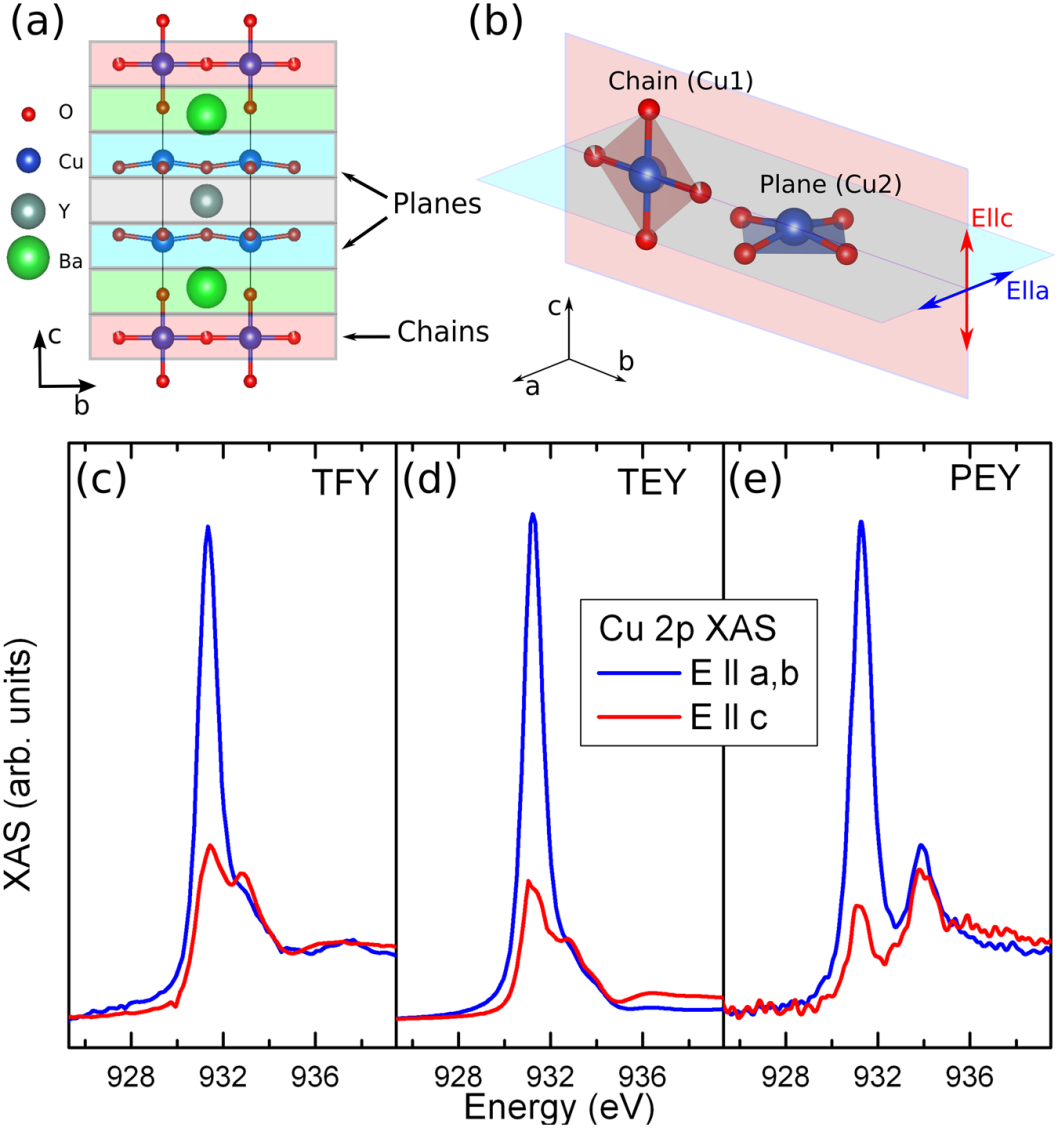
(**a**) Crystal structure of YBa_2_Cu_3_O_7−*δ*_ showing characteristic structural features such as the Cu chains and planes. (**b**) XAS geometry with respect to the YBCO structure and its different Cu sites (chain and plane). Note that in a twinned structure there is no clear difference between *a* and *b* directions. 2*p* XAS obtained from TFY (**c**), TEY (**d**) and PEY (**e**) measurements for in-plane (blue) and out-of-plane (red) polarized light. For clarity only the *L*_3_ edge is shown.

These reports however, deal with materials where a single resonant ionic species is considered that reconstructs electronically close to an interface. More complex systems like Fe_3_O_4_ or YBa_2_Cu_3_O_7−*δ*_ (YBCO) where the same ion exists within the crystal structure with different local symmetries and different valences will require a different approach to properly study modifications of electronic properties. To address this issue we have studied a film of the archetypical high temperature superconductor YBCO grown on SrTiO_3_ (STO). This system crystallizes with a layered structure, where Cu ions are located at two different crystallographic sites, namely chains (Cu1) and planes (Cu2) (see Fig. 1(a,b)). Each of them with distinct local symmetries and well known anisotropic distribution of their 3*d* charge^7,31,32^. Here we apply RXR to this complex situation and therefore go one step further towards monolayer resolution and site selectivity. To this end, based on our previous work^25^, we have developed an analysis scheme of RXR where the atomic structure of the material is taken into account by “slicing” it into atomic planes with characteristic anisotropic scattering properties. Using RXR at the Cu *L*_2,3_ edges, the stacking sequence and atomic termination of a YBCO thin film is determined. Our results show that at the surface the CuO chain layer has an electronic state different from that of the bulk and that it influences *only* the first CuO_2_ plane which is closest to the surface. Our method allows to determine structural information such as interface terminations and stacking of atomic layers. But more importantly, it enables to extract depth-resolved spectroscopic information with monolayer resolution which discriminates between different Cu sites, thus further enhancing the capability of the technique to study novel electronic phenomena at surfaces and interfaces.

## Results

### X-ray absorption spectroscopy

The crystal structure of YBCO is shown in Fig. 1(a). In this structure Cu ions are located at two distinct crystallographic sites, namely chains and planes, with very different local symmetries. We have measured the Cu 2*p* XAS spectra on a 6 unit cells (u.c.) thick YBCO film grown on STO (see methods section). The measurements were done for different alignments of the photon electric field **E** with respect to the YBCO lattice orientations in order to obtain spectroscopic information from the different Cu sites (cf. Fig. 1(b) and Methods).

Figure 1(c,d) shows the Cu 2*p* polarization dependent XAS spectra of the YBCO film measured in the total fluorescence yield (TFY) and total electron yield (TEY) modes, respectively. The observed lineshapes show typical absorption spectra of hole doped YBCO^7,31,33^. For ${\bf{E}}\parallel ab$ (blue lines in Fig. 1(c–e)), the strong peak at 931.3 eV corresponds to holes in the $3{d}_{{x}^{2}-{y}^{2}}$ orbital of the Cu (3*d* ^9^) in the planes^34^. The high energy shoulder at 932.8 eV, more pronounced in TFY (cf. Fig. 1(c)), has two contributions. The first corresponds to ligand holes ($3{d}^{9}\underline{L}$) with the same $3{d}_{{x}^{2}-{y}^{2}}$ symmetry in the planes, while the second contribution comes $3{d}^{9}\underline{L}$ ligand holes of the chain Cu with $3{d}_{{y}^{2}-{z}^{2}}$ orbital character^7,32^ (see crystal structure in Fig. 1(a,b)).

For an untwinned film, the Cu chains run along the *b* direction hence the lineshapes of ${\bf{E}}\parallel a$ and ${\bf{E}}\parallel b$ are different. For instance for ${\bf{E}}\parallel b$ a shoulder above 931.3 eV would be observed, due to the CuO chains running along this direction, which would be absent when ${\bf{E}}\parallel a$ ^32^. However, in a normal incidence geometry we do not observe any significant difference when rotating **E** by 90° in the *ab*-plane, hence, we can conclude that the film is twinned, as expected.

The ${\bf{E}}\parallel c$ lineshape (red lines in Fig. 1(c–e)) shows two peaks at 931.4 eV and 932.8 eV coming from holes in the $3{d}_{{y}^{2}-{z}^{2}}$ orbitals and $3{d}^{9}\underline{L}$ ligand holes of the chain Cu, respectively. Additionally, $3{d}_{{x}^{2}-{y}^{2}}/3{d}_{3{z}^{2}-{r}^{2}}$ hybridized states of the plane Cu also contribute to the lineshape although this contribution is small^34^.

A very different lineshape is observed in the partial electron yield (PEY) spectra (cf. Fig. 1(e)). Here, a second peak at 933.9 eV is clearly distinguishable for both ${\bf{E}}\parallel ab$ and ${\bf{E}}\parallel c$, which is attributed to monovalent Cu chains^32,35^. Such lineshape has been observed in oxygen depleted YBa_2_Cu_3_O_6_^7,31–33^. Due to the surface sensitive character of PEY as compared to TEY (cf. methods section), we can conclude that an oxygen depleted region exists at the film surface. In the rest of the film the Cu shows typical signatures of hole doped YBCO.

### Resonant x-ray reflectometry

**Figure 2:**
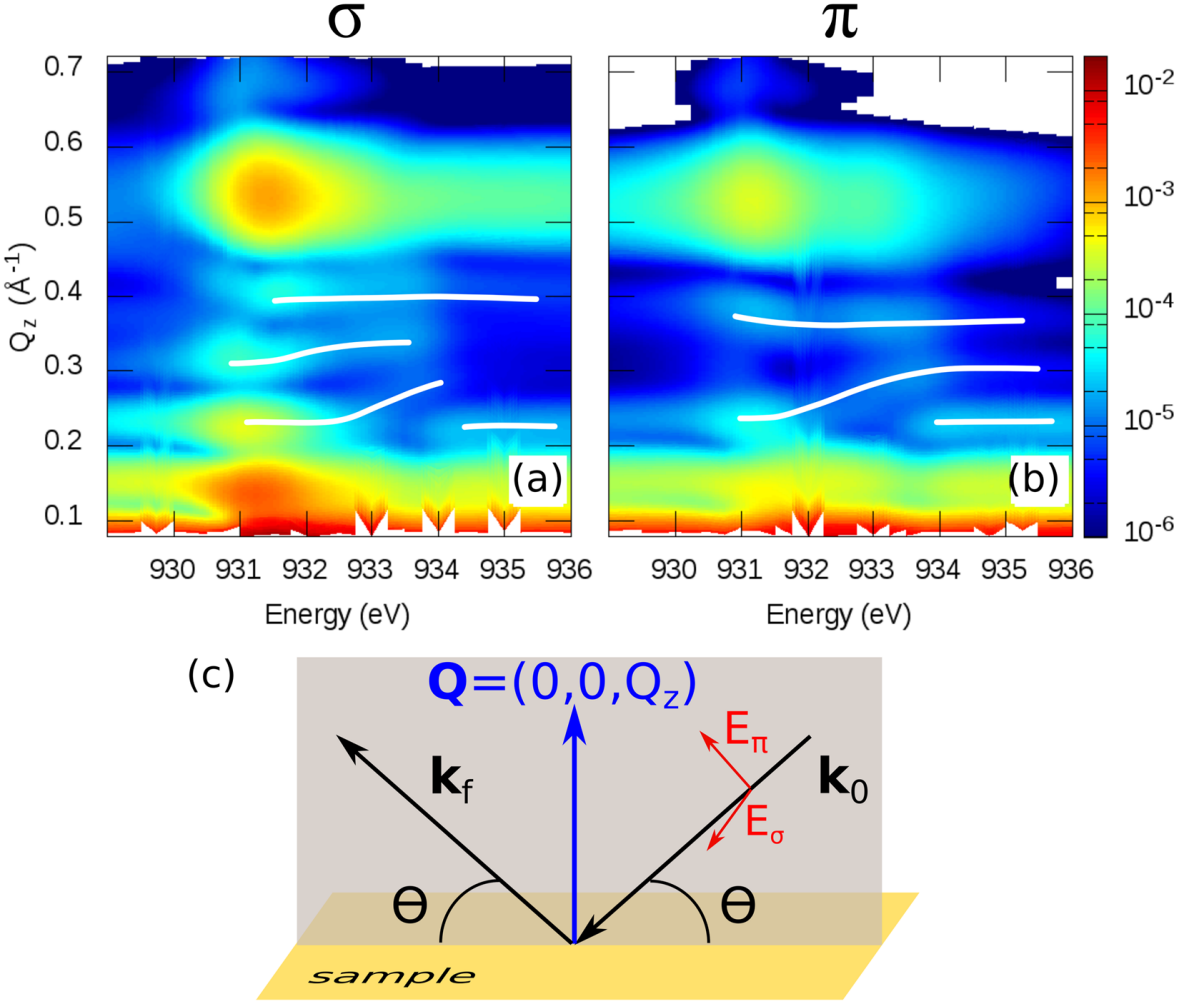
Experimental resonant x-ray reflectivities measured at energies close to the Cu *L* edges. The data was collected using *σ* (**a**) and *π* (**b**) polarized light. (**c**) Scattering geometry of a reflectivity experiment. For the data collection a set of *θ*–2*θ* scans has been measured using both polarizations at various photon energies across the Cu edge. White lines in (**a**,**b**) are guides to the eye and show the energy dependence of characteristic maxima. For clarity only the *L*_3_ edge is shown.

In Fig. 2(a,b) a representative part of the experimental RXR data is presented as Q_*z*_ vs. *hν* intensity maps. Q_*z*_ refers to the *z* component of the momentum transfer vector **Q** shown in Fig. 2(c). These maps were measured at energies close to the Cu *L*_2,3_ edges using *σ* and *π* polarized light, respectively. The maps show in addition to the characteristic thickness oscillations (Kiessig fringes), the (001) Bragg reflection present at Q_*z*_ = 0.54 Å^−1^ corresponding to a *c* lattice parameter of 11.635 Å (in bulk *c* = 11.68 Å^36^), which is in agreement with the expected in-plane tensile strain induced by the substrate.

As can be observed in Fig. 2 there is a strong energy and polarization dependence of the reflected intensities. For instance, the white lines drawn in the figure illustrate regions in the RXR-profile with particularly prominent intensity variations as a function of the incident photon energy *hν* and polarization. These changes are especially obvious at *hν* > 931.3 eV and result from strong changes of the materials refraction index *n*(*ω*) = 1 − *δ* + *iβ*. The later depends on the Cu scattering factors *f* ′(*ω*) and *f* ″(*ω*), which both change rapidly at the Cu *L*_2,3_-edge.

For the further analysis of the measured reflectivities, it is therefore important to determine the scattering factors *f *′(*ω*) and *f* ″(*ω*) which define the materials optical constants *δ* and *β*. Details of how the scattering factors for the different Cu sites were determined are discussed in the following section.

#### Determination of optical constants

In YBCO the Cu ions are located at two distinct lattice sites belonging to the chains (Cu1) and planes (Cu2) (cf. Fig. 1(b)). These sites have very different local symmetries yielding strong electronic anisotropies revealed by the polarization dependent Cu *L*_2,3_ XAS spectra in Fig. 1. In order to implement these local anisotropies a tensorial description of the resonant scattering length is necessary in order to describe the resulting polarization dependent scattering. We therefore introduce atomic scattering tensors for the Cu sites of the form

1 $${\hat{f}}_{l}(\omega )=(\begin{array}{ccc}{f}_{xx}(\omega ) & {f}_{xy}(\omega ) & {f}_{xz}(\omega )\\ {f}_{yx}(\omega ) & {f}_{yy}(\omega ) & {f}_{yz}(\omega )\\ {f}_{zx}(\omega ) & {f}_{zy}(\omega ) & {f}_{zz}(\omega )\end{array})$$

with ${f}_{mn}(\omega )={f}_{mn}^{^{\prime} }(\omega )+i{f}_{mn}^{^{\prime\prime} }(\omega )$, where the *x*, *y* and *z* coordinate refer to the *a*, *b* and *c* crystallographic orientations, respectively. The scattering tensor encodes information about the local electronic structure of the resonant scatterer, i.e., magnetization direction as well as the local coordination and interactions with the ligands^37^. Using the approach of Hawthorn *et al*.^32^ and Nücker *et al*.^7^, the scattering tensors for Cu1 and Cu2 were determined from our experimental polarization dependent TEY-XAS spectra described above.

Considering first the Cu in the planes (Cu2), as discussed by Nücker *et al*.^7^, the ${f}_{xx}^{^{\prime\prime} }={f}_{yy}^{^{\prime\prime} }$ term for the plane Cu can be determined from the difference between ${\bf{E}}\parallel c$ and ${\bf{E}}\parallel ab$ XAS spectra. This can be better understood by looking at Fig. 1(b). The XAS signal obtained when measuring with ${\bf{E}}\parallel c$ is vastly governed by absorption from the chain Cu (Cu1). Whereas, for ${\bf{E}}\parallel ab$ the absorption comes from both chain and planes. Now, since ${f}_{zz}^{^{\prime\prime} }={f}_{yy}^{^{\prime\prime} }$ for the chains and due to the fact that the sample is twinned, the signal obtained by subtracting the ${\bf{E}}\parallel c$ and ${\bf{E}}\parallel ab$ XAS will, in a good approximation, yield the spectra which come only from the planes.

**Figure 3:**
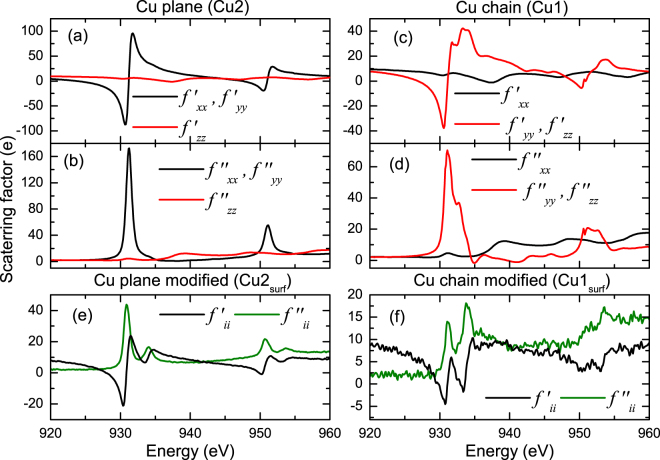
Real (*f* ′) and imaginary (*f *″) part of the diagonal terms of the atomic scattering tensor for Cu2 (**a**,**b**) and Cu1 (**c**,**d**). (**e**,**f**) Shows those taken for the topmost plane (Cu2_*surf*_) and chain (Cu1_*surf*_) sites.

Due to the impossibility to fully disentangle Cu1 and Cu2 contributions to the ${\bf{E}}\parallel c$ XAS spectra. The weak Cu2 ${f}_{zz}^{^{\prime\prime} }$ term (red line in Fig. 3(b)) was determined using XAS data measured on bulk La_1.85_Sr_0.15_CuO_4_^34^ (LSCO), which only contains CuO_2_ planes. Note, however, that using this is an approximation, the local Cu environment is different in LSCO and YBCO. Yet, these differences are rather small and the corresponding scattering amplitudes in the *z*-direction (out of the CuO_2_ plane) are weak. As a result, the present approximation introduces only minor errors.

For the chain Cu (Cu1), the ${f}_{zz}^{^{\prime\prime} }={f}_{yy}^{^{\prime\prime} }$ term was obtained as follows: First we have normalized the ${\bf{E}}\parallel c$ XAS signal from 0 below the edge, to 1 after the edge. Due to the stoichiometry of YBCO, 1/3 of this signal is contributed by Cu1 and 2/3 by Cu2. In order to obtain reliable values of the Cu1 scattering factors we need first to subtract the signal contributed by the two Cu2 sites. To do so we have assumed that each Cu2 contributes a signal given by a step function at the *L*_2,3_ edge jump. This is justified by the Cu2 local symmetry, which has no states to excite into along the *z* direction^7,34^. Therefore, the Cu1, ${f}_{zz}^{^{\prime\prime} }={f}_{yy}^{^{\prime\prime} }$ was obtained by subtracting 2/3 of the step jump from the normalized ${\bf{E}}\parallel c$ XAS.

For the calculations of the reflectivity we have made the following assumptions: First, since the film is twinned, as concluded from the XAS, we set ${f}_{xx}={f}_{yy}=\overline{f}$ for Cu1. Second, during the scattering process there is no coherence between twin domains. Third, in the absence of long range magnetic order or external magnetic fields the system can be described by a scattering tensor with zero off-diagonal contributions^38^.

Figure 3 shows the real and imaginary parts of the diagonal terms of the scattering matrices for Cu2 ((a) and (b)) and Cu1 ((c) and (d)). The obtained line-shapes are in close agreement with the reports by Hawthorn *et al*.^32^. For all the other ions, including those in the STO substrate, the atomic scattering factors were assumed to be isotropic, i.e., *f*_*xx*_ = *f*_*yy*_ = *f*_*zz*_ with all off-diagonal terms equal to zero, and their values were taken from tabulated data^39^.

#### Film structure determination

**Figure 4:**
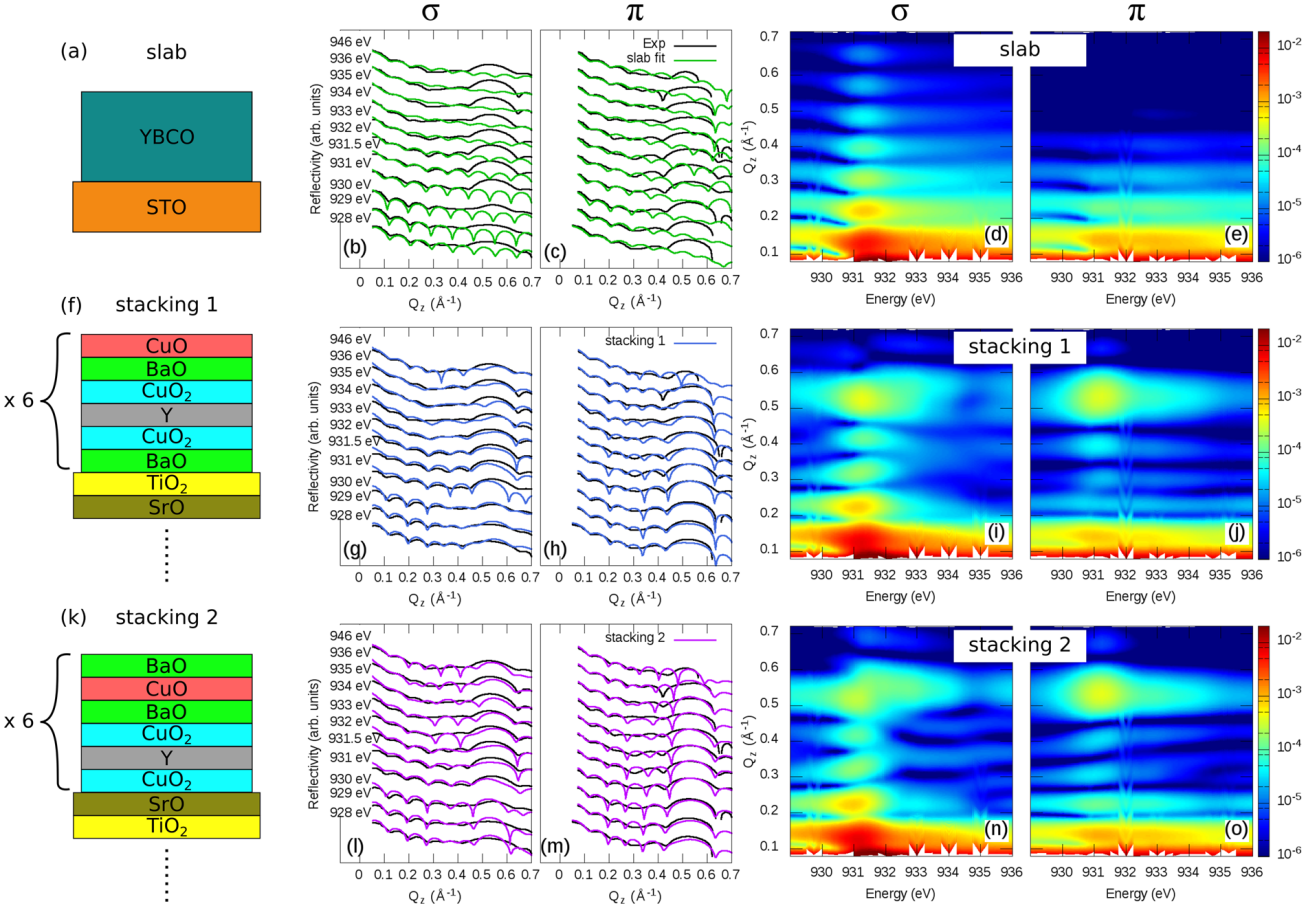
Structural models and fitted reflectivities. The upper, middle and lower rows show the fits corresponding to the (**a**) “slab”, (**b**) “*stacking 1*” and (**c**) “*stacking 2*” structural models, respectively. The total film thickness is 6 u.c., i.e., 6 × (6 atomic layers) = 36 atomic layers. Black lines in (**b**,**c**,**g**,**h**,**l**,**m**) show experimental data. Color plots are Q_*z*_ vs. *hν* intensity maps calculated using the corresponding structural models. Greek letters indicate the polarization of the light used for the measurement/calculation.

The first step in our RXR analysis is to establish a structural model of the YBCO film, i.e., to determine the layer thicknesses, interfaces and stacking sequences. Due to the presence of the Bragg reflection, analysis of the reflectivity in terms of homogeneous slabs as sketched in Fig. 4(a) is not possible. As shown in Fig. 4(b,c), a calculation within the slab approach (green curves) is incapable to reproduce the Bragg peak observed in the experimental reflectivities (black curves). This is more obvious when looking at the calculated intensity maps in Fig. 4(d,e), where only the Kissig fringes are observed in contrast to the experimental maps in Fig. 2. This is because a slab approximation considers only reflection of the x-rays at surfaces and interfaces of the materials conforming the heterostructure. It completely neglects the atomic crystal structure of the heterostructure and as such cannot reproduce the observed Bragg peak, which is a direct consequence of the crystalline structure of the material.

**Figure 5:**
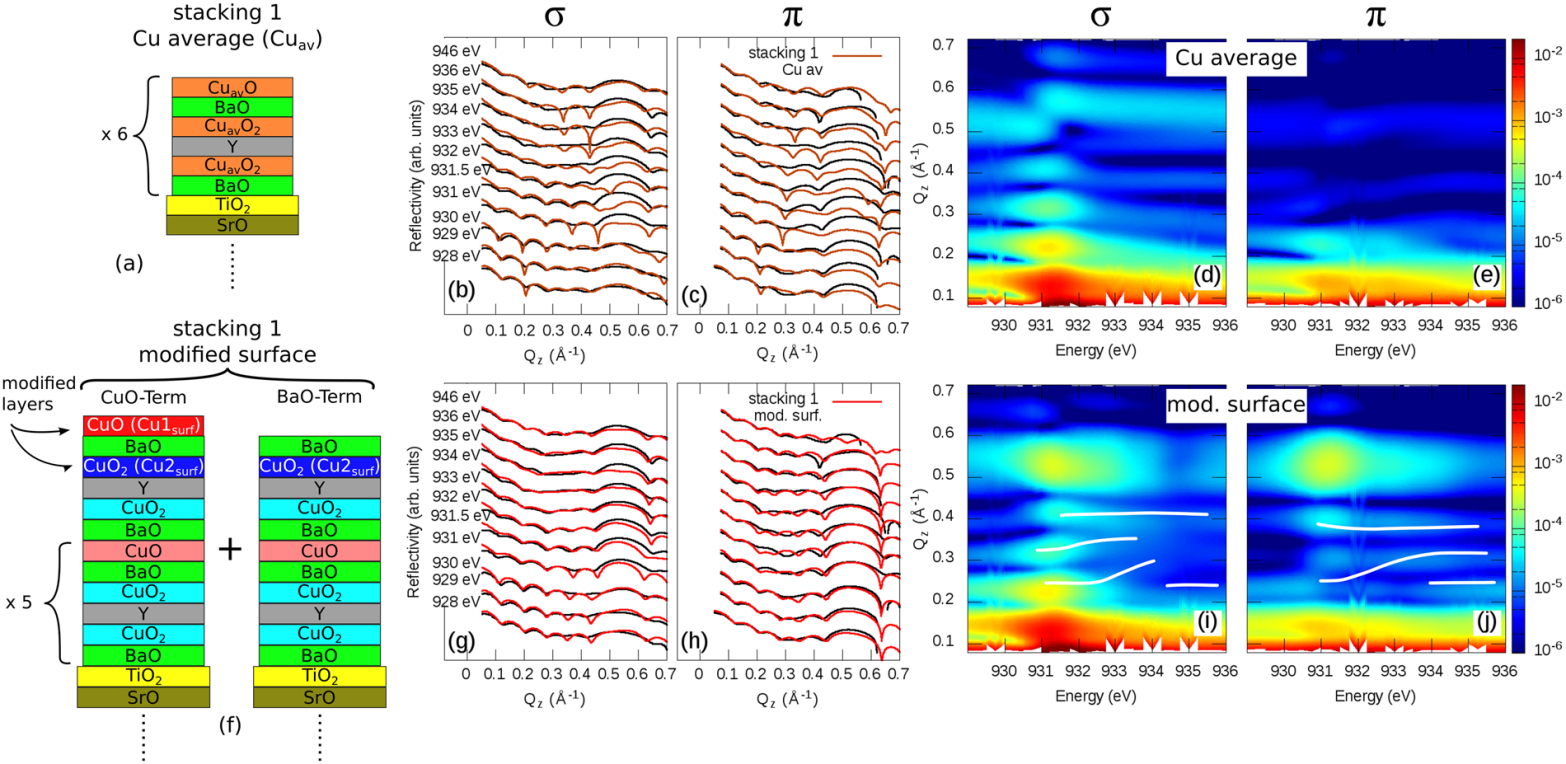
Structural models and fitted reflectivities. The upper row shows the fits using *stacking 1* and an average Cu-scatterer “Cu average” (explanations in the text). Lower row show the fits for the *stacking 1* including the modified surface. (**f**) Shows the two CuO and BaO terminations and the location of the reconstructed Cu sites at the surface. Note that for the model shown in (**f**) the total film thickness is still 6 u.c. Here we have 5 bulk-like unit cells and one surface u.c. which is reconstructed. So for CuO-Term there are 5 × (6 atomic layers) + 6 atomic layer = 36 atomic layers. Whereas for BaO-Term there are 5 × (6 atomic layers) + 5 atomic layers = 35 atomic layers. Black lines in (**b**,**c**,**g**,**h**) show experimental data. Color plots are Q_*z*_ vs. *hν* intensity maps calculated using the corresponding structural models. Greek letters indicate the polarization of the light used for the measurement/calculation (cf. Fig. 2(c)). White lines in (**i**,**j**) are a guide to the eye and show the energy dependence of the thickness oscillations maxima. These lines are exactly the same as in Fig. 2(a,b).

To overcome this problem we have adopted the “atomic slices” approach which was developed in ref.^25^. In this approach the YBCO unit cell has been subdivided into thinner slabs (slices) corresponding to its atomic planes. The optical constants of each atomic slice have been defined according to the stoichiometry of the corresponding lattice plane. As described in our previous work^25^, when using the slice approach to reflectivity, a good way to model the transition from one layered material into another is using an envelope error function of the form ${\rm{erf}}(\mu ,\,\sigma )=(1/\sigma \sqrt{2\pi })\,{\int }_{-\infty }^{z}\,\exp -({\mu }^{2}/2{\sigma }^{2})\,d\mu$. With *μ* = (*z*_*i*_ − *z*_0_), where *z*_*i*_ is an integer number defining the location of an atomic layer of the material close to the interface, *z*_0_ is an integer number defining the layer position where the interface is located and *σ* defines the width of the transition, i.e., the interface roughness.

During the analysis of the reflectivities, a surface layer was introduced accounting for possible organic and other contaminants that might adhere to the film surface. Its optical constants were taken as those from carbon. In this way, we have defined three interfaces namely: STO/YBCO, YBCO/C and C/vacuum. For each of these, the interface location *z*_0_ and roughness *σ* were fitted. Note that the layer thickness is given by the relative positions between interfaces, whereas the *z*_0_ location defines the atomic termination at the interface. In addition to these parameters, an overall scaling factor, used to match the calculated and measured intensity scales, was also fitted yielding a total of seven fitting parameters. All reflectivities including both *σ* and *π* polarizations were fitted simultaneously. This involves fitting the whole energy range hence, ensuring a self consistent result free from local bias given by particular features at a particular energy.

The fitting was done in two steps, first an evolutionary algorithm^40^ was used. This ensures that the fit is completely unbiased by evaluating a large region of possible configurations in parameter space. The resulting best fit was then followed by a simplex least square reduction algorithm. The quality of the fit was judged by a qualitative comparison of fitted vs. experimental data, and quantitatively by determining the deviation between experimental and calculated reflectivities by means of the *χ* ^2^ error (see Table 1).

**Table 1:** Fit error *χ* ^2^ for different stackings, terminations (cf. Figs 4 and 5) and electronic models: Bulk-like, refers to the case where no changes in electronic properties with respect to the bulk are considered.

Model	*χ* ^2^
Stacking 1 CuO-Term + BaO-Term (mod. surf)	9.11
Stacking 1 CuO-Term + BaO-Term (Bulk-like)	10.69
Stacking 1 CuO-Term (mod. surf)	10.84
Stacking 1 CuO-Term (Bulk-like)	12.10
Stacking 1 BaO-Term (mod. surf)	12.99
Stacking 1 BaO-Term (Bulk-like)	12.46
Stacking 1 (Cu average)	15.16
Stacking 2 BaO-Term (Bulk-like)	20.48

Modified surface (mod. surf) here the Cu1_surf_ and Cu2_surf_ have distinct but isotropic $\hat{f}(\omega )$. Cu average refers to the case where ${\hat{f}}_{{\rm{Cu}}1}={\hat{f}}_{{\rm{Cu}}2}={\hat{f}}_{{{\rm{Cu}}}_{{\rm{av}}}}$.

Figure 4(g–j), shows the fit results with the lowest *χ* ^2^ value, which implement the atomic slices approach using the site specific Cu1 (chains) and Cu2 (planes) scattering tensors (cf. Fig. 3(a–d)). This model nicely describes the observed Bragg peak, thus yielding an obvious improvement over the slab model. Moreover, from the fit, the best description of the experimental data is given by a film structure where the YBCO surface is YBCO-BaO-CuO terminated and the STO/YBCO interface has a STO-TiO_2_-BaO termination as shown in Fig. 4(f) (*stacking 1*). Moreover, from the fitted maps (Fig. 4(i,j)) one can see how the strong polarization dependence emerging from the anisotropic scattering of the different Cu sites is well retrieved by implementing the scattering tensor. In strong contrast, assuming a homogeneous slab, no Bragg peak can be reproduced and no detailed structural information, out of the YBCO total thickness, can be extracted.

In order to put our results in perspective and to better understand their implications, let us discuss two additional models:

(i) Let us assume a film structure with the same thickness as *stacking 1* but different stacking of the atomic planes, thus with different interface terminations. This structure is shown in Fig. 4(k) (*stacking 2*), here the surface termination is YBCO-CuO-BaO and the STO/YBCO interface is given by SrO-CuO_2_, which is known to be also possible^41^. Figure 4(l–o) show the corresponding calculated reflectivity. Qualitatively from the figures one can judge that this structure does not fully describe the data. Moreover, it has a *χ* ^2^ value which is about 70% larger than that of *stacking 1* (20.48 vs. 12.10 in *χ* ^2^). This nicely demonstrates that the changes in the relative phases of the resonant scatterers, which are caused by changing the stacking, are clearly visible in the RXR signal. This allows to easily discern between different atomic stacking sequences. Furthermore, considering that the only difference between *stacking 1* and *2* is just a shift along *z* of the stacking by one atomic plane and that such difference can be discerned, this result demonstrates the remarkable monolayer resolution of RXR.
(ii) To demonstrate the importance of implementing two $\hat{f}$-tensors for Cu1 and Cu2, in Fig. 5(a–e) we show calculated RXR for an average $\hat{f}$-tensor used for both the Cu1 and Cu2 sites. More specifically, this model neglects the differences between Cu1 and Cu2 and describes both sites by an average $\hat{f}$-tensor (Cu_*av*_) that has been obtained assuming that its in-plane *f*_*xx*_ = *f*_*yy*_ and out-of-plane *f*_*zz*_ components correspond to the “as is” ${\bf{E}}\parallel ab$ and ${\bf{E}}\parallel c$ experimental XAS, respectively. As can be observed in Fig. 5(b,c) (blue lines) and maps (d) and (e), this “Cu average” model fails to properly describe the experiment, even using an atomic slice approach. This is particularly evident by looking at the *π* channel (Fig. 5(e)) where the Bragg peak is barely observable. Hence, a realistic description of the experimental data requires a model where each Cu site is explicitly described together with its local anisotropies.

The following analysis will therefore be based on the atomic slices approach using the two different $\hat{f}$ tensors for Cu1 and Cu2 and *stacking 1*. However, we also found that *stacking 1* with the topmost CuO layer missing, hence BaO terminated (Fig. 5(f)), also provides an acceptable structural model. We therefore assume that both surface terminations are present, which is in perfect agreement with earlier STM results^14,22,42,43^. Also the TiO_2_-BaO termination at the STO/YBCO interface agrees to what has been observed with TEM^13^.

The structural model used in the following analysis hence consists of CuO (CuO-Term) and BaO (BaO-Term) terminated domains of *stacking 1*. This calculation assumes that these domains scatter light incoherently. In this way we can express the total intensity reflectivity as *I*_Tot_ = (1 − *x*)*I*_CuO–Term_ + *x* *I*_BaO–Term_, where *x*, is a factor that accounts for relative domain population. By testing different values of *x* we have found that *χ* ^2^ of *stacking 1* is considerably reduced by about 12% for *x* = 0.5 (cf. Table 1). Our analysis therefore implies that our YBCO film surface consists of *stacking 1* with an equal population of both CuO and BaO surface terminations.

#### Surface reconstruction

In the second step of our RXR analysis we now use the structural model established above to extract depth resolved information about electronic properties and their variations at the interfaces.

So far we have assumed that both chains and planes have the electronic properties of the bulk throughout the whole film. In reality, this “bulk-like” scenario is rather unlikely close to the surface, scanning tunneling spectroscopy (STS) and angle-resolved photoemission spectroscopy (ARPES) experiments have provided evidence of distinct electronic properties of Cu at the surface. Moreover, electronic modification can also take place at the interface with the substrate.

During the data analysis, several models assuming a modification of electronic properties somewhere in the film were examined using the available input from XAS. For instance, the PEY reveals a modified surface scenario. Implementing this into our model yielded a much better agreement with the experiment and a further 15% reduction of *χ* ^2^ from 10.69 to 9.11. Figure 5(f) shows the model with the lowest *χ* ^2^. It consists of a 6 u.c. YBCO film whose surface has equal population of domains with CuO and BaO terminations. For these structures, the topmost (surface) CuO and CuO_2_ layers have different electronic properties in comparison to the film bulk. The scattering tensor for the surface CuO (Cu1_s*urf*_) layer was constructed using the ${\bf{E}}\parallel c$ PEY spectra (cf. Fig. 1(e)) assuming an isotropic scattering tensor, i.e., *f*_*xx*_ = *f*_*yy*_ = *f*_*zz*_ (cf. Fig. 3(f)). Similarly for the modified Cu plane (Cu2_s*urf*_), its isotropic scattering tensor was built using the ${\bf{E}}\parallel ab$ PEY (cf. Fig. 1(e)).

Figure 5(g,h) show the experimental (black) and fitted (red) reflectivity curves using this modified model. As compared to the bulk-like (cf. *stacking 1* in Fig. 4(b,c)) and average Cu approaches (cf. Fig. 5(b,c)) the agreement is very satisfactory. Also the energy and polarization dependence of the maxima of the thickness oscillations are fairly well reproduced as shown in the intensity maps in Fig. 5(i,j). For instance, the oscillation maxima closely follow the energy dependence observed in the experiment (see white lines in the Fig. 5(i,j)).

Let us now discuss some details of this model. Our analysis yields that the film topmost atomic plane for the domain with *stacking 1* and CuO termination is the chain CuO layer. This surface layer has scattering factors reminiscent to that of oxygen depleted YBCO^7,31–33^, which is naturally expected due to loss of oxygen in the chains at the surface. Considering that for YBCO, the CuO chains are the charge reservoir for the superconducting CuO_2_ planes, it is also expected that changes in the CuO chain will affect the planes. Our results show that this is indeed the case. From the two CuO_2_ planes underneath the surface CuO layer *only* the one which is closest to the surface is found to be modified. A similar scenario is seen for the BaO terminated domain: here without the disrupted CuO layer, but only the modified CuO_2_ layer just beneath the BaO capping. Note that, although in each domain the topmost CuO_2_ plane its being covered with different layers, the Cu2_*surf*_ is the same for both of them. Thus implying that the electronic properties of the topmost CuO_2_ layer is the same in the two domains.

This change in the polarization dependent scattering of Cu2 in the “surface” plane clearly indicates an orbital reconstruction in which part of the holes, normally constrained to the *xy*-plane, are relocated in the *z*-direction. Similar situations have been observed at other cuprate/perovskite interfaces^8,11,12^ and has been ascribed to hybridization of $3{d}_{3{z}^{2}-{r}^{2}}$ orbitals of the Cu and the TM ion in the perovskite structure via apical oxygen. In the case of LCMO/YBCO^8^, the polarization dependent XAS is remarkably similar to that shown in Fig. 3(e), which is indicative of interface electron doping.

In contrast to the case of manganite/cuprate interfaces, where electron transfer from the manganite to the YBCO at the interface is commonly observed^44–47^, the orbital reconstruction here reported is not due to a charge transfer between Mn and Cu across an interface but to a combination of local symmetry breaking and a modification of the electron count at the surface due to the oxygen deficient CuO surface chains.

## Discussion and Conclusions

Still, after considering a surface modification, we cannot reproduce all details shown in Fig. 2(a,b). This can be attributed to the following two complications: Firstly, a close inspection of Fig. 5(i,j), reveals that although a good description of the *σ* reflectivities is obtained with the current model, this does not fully apply for *π* reflectivities. Due to geometrical reasons, reflectivities measured using *π* polarized light encode more information about electronic states which are pointing away from the film surface. Deviations for the *π* reflectivities therefore imply that for some of the sites the *f*_*zz*_ terms are not estimated well. This is more likely to happen for the modified ions at the surface since their real structure is unknown. Secondly, so far we have completely neglected any electronic modification at the substrate-film interface. The inclusion of the STO/YBCO interface is a major challenge, because of the complexity of YBCO and its many distinct and anisotropic Cu sites, e.g., at planes, chains and those sites which are electronically modified at the surface and interface. This results in a large number of additional parameters which cannot be determined reliably based on the measured data set. Nonetheless the fact that the favored model already captures the major features of the experimental RXR data, implies that the dominant effects are taken into account.

A way to overcome these limitations would be to augment the above analysis by state-of-the-art many-body calculations. Using for instance embedded cluster calculations^48^, realistic estimates for the *f* ′ and *f* ″ at the interfaces can be obtained and implemented into our modeling.

To summarize, the crystal structure stacking and surface termination of a 6 u.c. optimally doped YBCO ultra thin film was determined by means of RXR. Our results show that the film starts with a STO-BaO-CuO_2_-YBCO interface and at the surface it has two domains with different terminations, one is YBCO-BaO-CuO and the other YBCO-CuO_2_-BaO terminated.

Regarding the electronic properties of the YBCO film, our element and site selective approach reveals that the electronic properties of the two topmost Cu-containing layers differ markedly from those present deeper inside the film. Specifically, we find oxygen depleted CuO chain-layers at the surface, which are represented by Cu1_*surf*_ in our analysis. This oxygen depleted top layer results in an electron-doped CuO_2_-plane right underneath the surface that is described by Cu2_*surf*_ in our analysis. It is worth stressing that the electron doped region is very strongly confined to the surface, as only the CuO_2_-plane just below the oxygen-depleted chains is found to be affected significantly. For this topmost CuO_2_-plane the presented analysis further provides evidence for an orbital reconstruction of the Cu 3*d*-states, similar to the one observed at YBCO/LCMO interfaces.

The presented reflectivity results, showing a surface electron doped YBCO layer, are in contrast to those from ARPES where an anomalous hole overdoped surface state is observed^49–51^. However, the ARPES spectra were collected on a cleaved bulk sample with a complex disrupted surface termination, whereas our data corresponds to a film with a surface termination given by the growth conditions. Hence, these two types of experiments are not easily comparable. Still, it is quite remarkable that probing with a wavelength, which is multiple times larger than the atomic layer thickness, we are not only sensitive to the bulk features, but we are also able to extract surface information with atomic layer resolution.

The present work shows the potential of RXR to study element and site selective electronic properties of surfaces and buried interfaces being depth resolved and non-destructive. This technique is a promising and very suitable tool to study emergent novel physical phenomena at surfaces and interfaces of complex materials and their heterostructures.

## Methods

### Materials

An optimally doped YBCO thin film 6 unit cells (u.c.) thick was grown on a (001)-SrTiO_3_ (STO) substrate by means of Pulsed Laser Epitaxy. The details of sample preparation can be found in ref.^52^.

### X-ray Spectroscopy and Scattering

X-ray absorption spectroscopy and resonant reflectivity experiments were carried out at the UE46-PGM1 beamline of the BESSY II storage ring of the Helmholtz-Zentrum Berlin (HZB). These experiments were performed using the XUV diffractometer and taking advantage of the novel fast continuous-mode motor scans that allow to acquire XAS and reflectivity (*θ*–2*θ* from 0° to 170°) data within minutes. The XAS was collected in the total electron yield (TEY) and total fluorescence yield (TFY) modes.

TFY is a bulk sensitive probe and collects the photons that are emitted by the sample after light illumination. TEY on the other hand measures the drain current from the photoelectrons that escape the sample after light irradiation. Since the electrons probing depth at the Cu *L* edges in YBCO is about 50 Å (4.3 u.c.’s)^53^, TEY is considered a surface sensitive probe.

Additional partial electron yield (PEY) measurements were carried out at beamline UE52 of the HZB. During collection of the PEY a mesh is placed before the detector so that only electrons with high kinetic energy can pass. In this way only electrons coming from the topmost layers of the material are measured. Hence, PEY is much more surface sensitive than the TEY thus yielding spectroscopic information of the film surface.

Both XAS and RXR experiments were performed using linearly *π* and *σ* polarized light at photon energies close to the Cu *L*_2,3_ edges. *π* and *σ* designate a polarization parallel and perpendicular to the scattering plane (cf. gray plane in Fig. 2(c)), respectively.

The X-ray absorption profiles were measured with *σ*- and *π*-polarization of the incident light at an angle of incidence of *θ* = 30° and 90°. Although for *σ*-polarization the measured intensity corresponds directly to ${\bf{E}}\parallel x$, the intensity for ${\bf{E}}\parallel z$ is deduced by performing the following geometrical correction *I*_*z*_ = *I*_*π*_/cos^2^ *θ* − *I*_*x*_ tan^2^ *θ*, with *I*_*π*_ being the measured intensity with *π*-polarization^54^. In this way one can extract absorption spectra where the photon electric field **E** aligns parallel and perpendicular to the film surface, i.e., **E** ⊥ *c* and ${\bf{E}}\parallel c$ of the YBCO, respectively.

The geometry of a specular reflectivity experiment is depicted in Fig. 2(c). Here an incoming x-ray beam, with wave vector **k**_0_ and *σ* or *π* polarization, hits the sample surface with an incidence angle *θ*. The intensity of the specularly reflected x-ray leaving the sample at an exit angle equal to *θ* is measured as a function of *θ*. The specular reflectivities (*θ*–2*θ* scans) were measured as a function of the *z*-component of the momentum transfer vector **Q** = (0, 0, Q_*z*_) ranging from 0.1 to 0.7 Å^−1^. The collected data set consists of 46 reflectivities per polarization, measured on and off resonance. The energy step close to the Cu *L*_2,3_ edges was 0.25 eV yielding a total of 19844 data points. All experiments were done at room temperature.

### Analysis of RXR data

The optical constants of the materials under study were constructed as described in refs^24,25,30^. First the measured XAS spectra are normalized to tabulated scattering factors^39^. Subsequently a Kramers-Kronig (KK) transformation is performed to obtain the real part *f* ′ of the scattering factor. For the KK calculation, an integral approach is chosen where an analytic expression is obtained by linearly interpolating successive values of the scattering factor *f* ″ in energies between E_*i*_ and E_*i*+1_, (cf., ref.^55^).

Analysis of the reflectivities were performed with the software ReMagX^26^. This program uses a dynamical approach where the one dimensional Maxwell equations are solved exactly within the model.

### Data availability

The authors declare that the data supporting the findings of this study are available from the corresponding author on reasonable request.
